# Supplementary material for: Synergic Effect of Brachyspira hyodysenteriae and Lawsonia intracellularis Coinfection: Anatomopathological and Microbiome Evaluation
Source: Animals (Basel). 2023 Aug 13;13(16):2611. doi: 10.3390/ani13162611 (PMC10451556; doi:10.3390/ani13162611)
Supplement: Supplementary file 1 [file animals-13-02611-s001.zip › animals-2511397-supplementary.pdf]

**Supplementary**

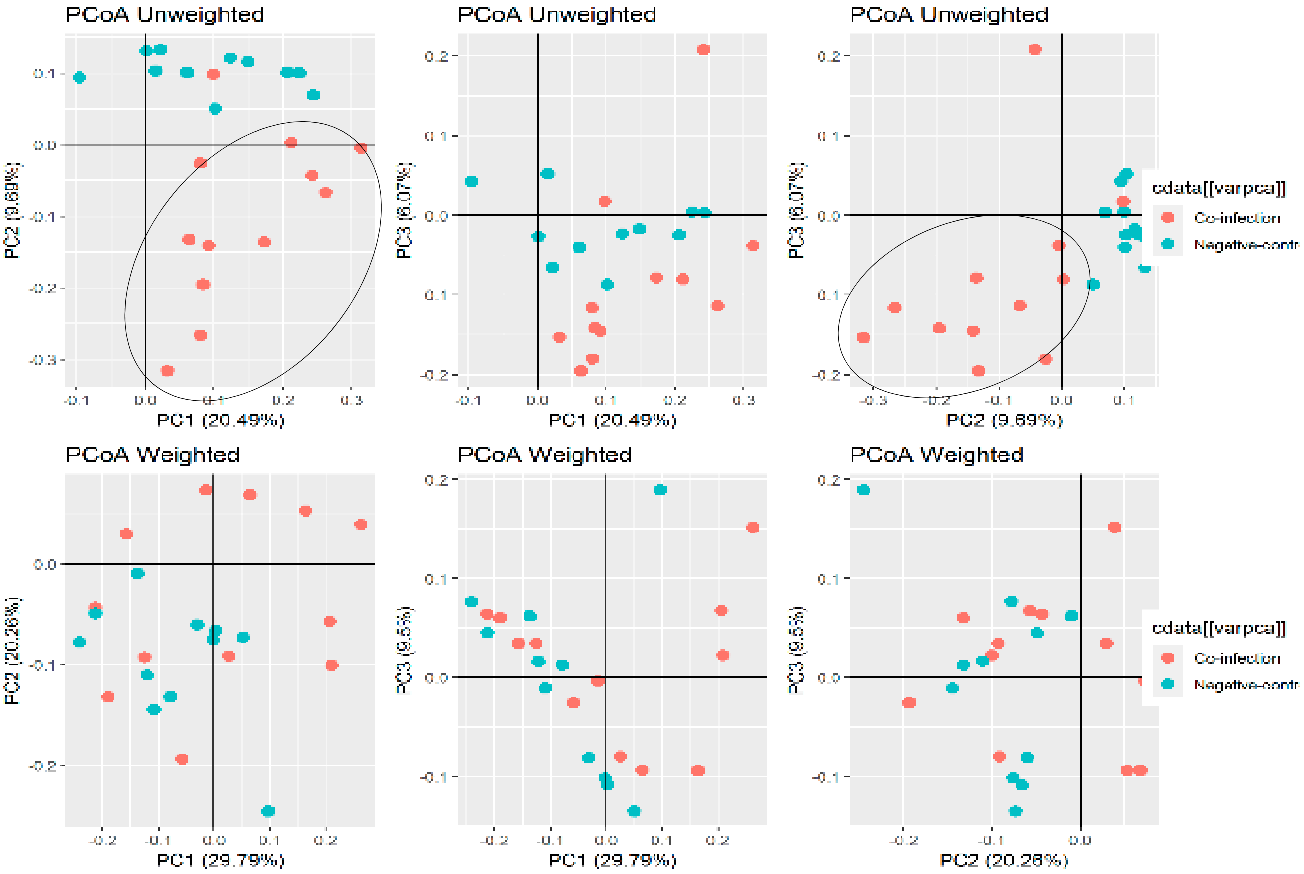


**Figure S1:** Principal coordinate analysis. The colors correspond to the groups made by Unifrac associated to fecal microbial communities. The beta diversity distance matrix was calculated to generate the diversity plot of swine microbiota samples at 21 days post infection comparing the coinfection and negative groups. It is possible to observe in the circles the formation of a close cluster in the coinfection group.

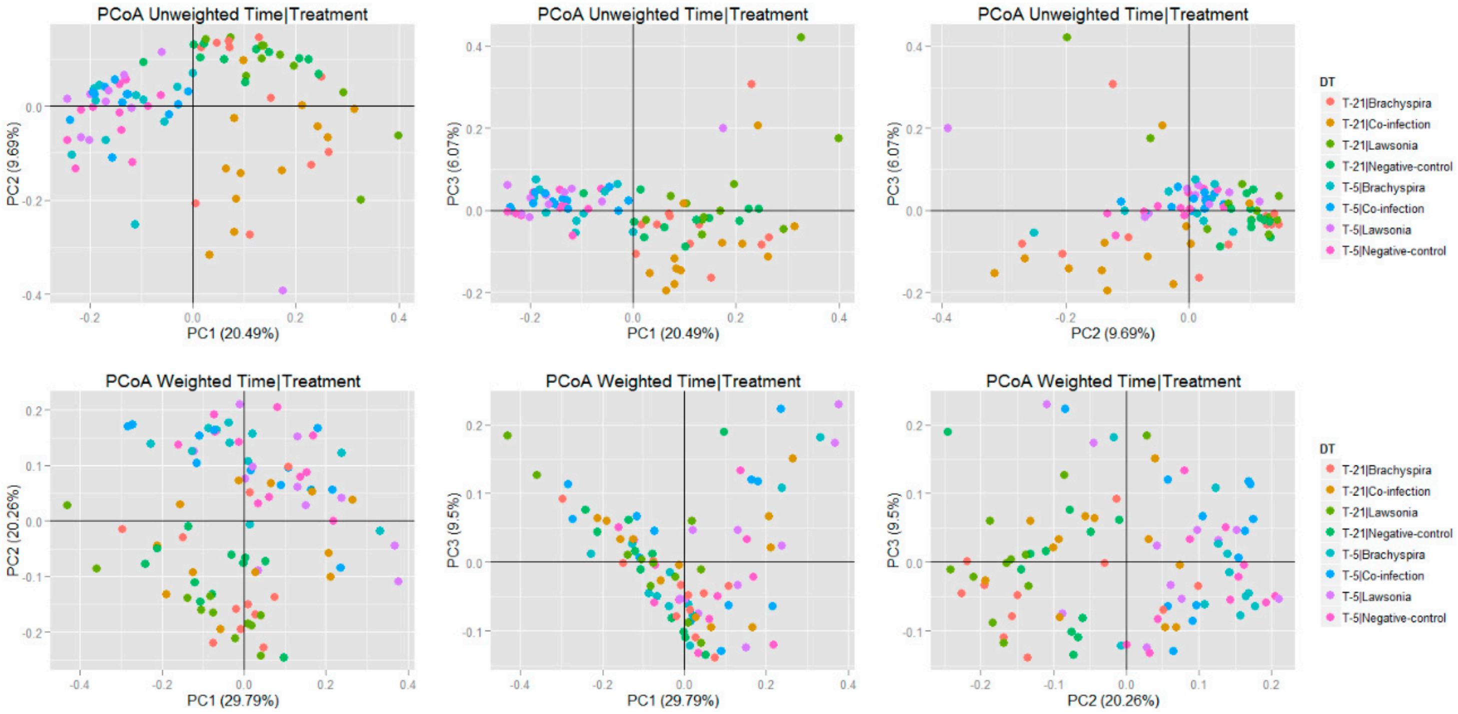


**Figure S2**: Principal coordinate analysis. The colors correspond to the groups made by Unifrac associated to fecal microbial communities. The beta diversity distance matrix was calculated to generate the diversity plot of swine microbiota samples at- 5 and 21 days post infection comparing the groups.

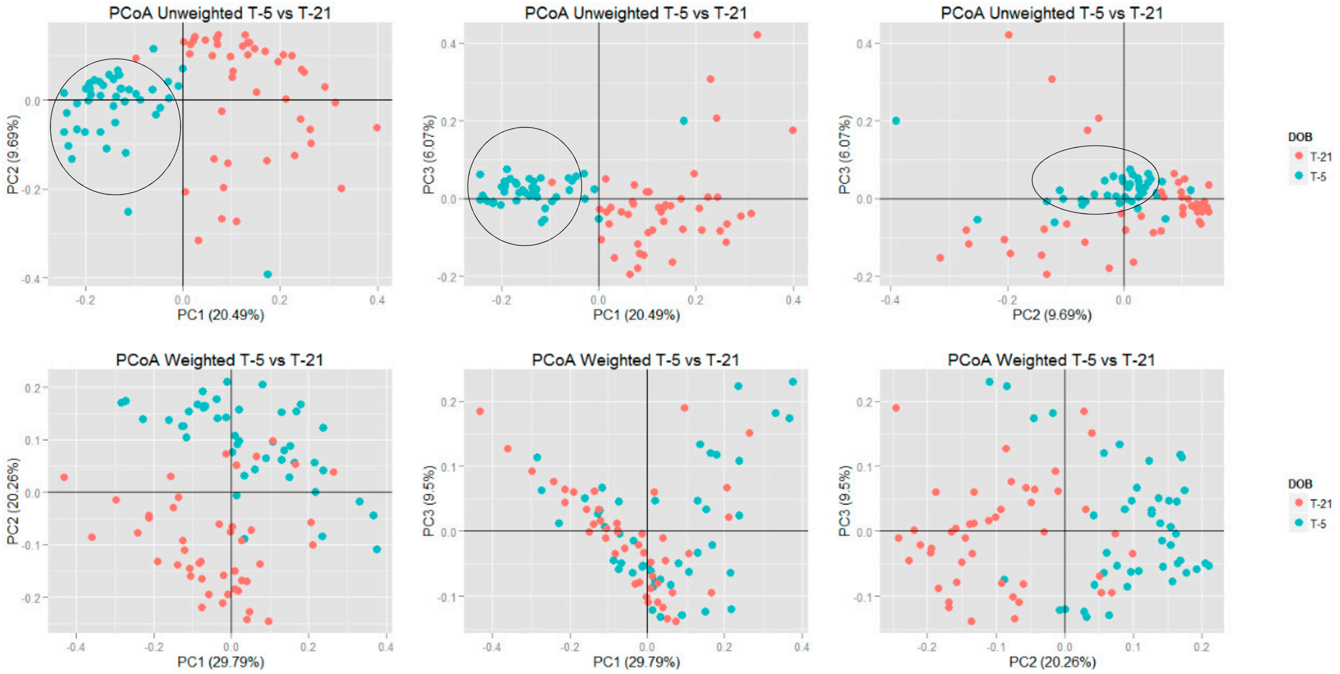


**Figure S3:** Principal coordinate analysis. The colors correspond to the groups made by Unifrac associated to fecal microbial communities. The beta diversity distance matrix was calculated to generate the diversity plot of swine microbiota samples at- 5 and 21 days post infection comparing the groups. It is possible to observe in the circles the formation of a close cluster in the coinfection group.

**Table S1**: Table of qPCR results comparing the fecal shedding, the letters "a,b" indicate statistical difference P≤ 0.05..

| *Lawsonia intracellularis* PCR | DPI | CO | BRA | LAW | NEG |
|---|---|---|---|---|---|
| | -5 | 0a | 0a | 0a | 0a |
| | 3 | 9,82x106a | 0a | 3,52x105a | 0a |
| | 6 | 1,80x105a | 0b | 1,06x106a | 0a |
| | 10 | 1,03x106a | 0b | 7,42x105a | 0b |
| | 12 | 1,16x106a | 0b | 1,24x106a | 0b |
| | 15 | 7,62x106a | 0b | 6,66x106a | 0b |
| | 18 | 3,45x106a | 0b | 3,35x106a | 0b |
| | 21 | 2,81x108a | 0b | 1,96x107a | 0b |

| *Brachyspira hyodysenteriae* PCR | DPI | CO | BRA | LAW | NEG |
|---|---|---|---|---|---|
| | -5 | $0^{a}$ | $0^{a}$ | $0^{a}$ | $0^{a}$ |
| | 3 | $0^{a}$ | $0^{a}$ | $0^{a}$ | $0^{a}$ |
| | 6 | $0^{a}$ | $0^{a}$ | $0^{a}$ | $0^{a}$ |
| | 10 | 3,82x10$^{3a}$ | 2,30x10$^{3ab}$ | $0^{b}$ | $0^{b}$ |
| | 12 | 2,50x10$^{6a}$ | 8,75x10$^{3a}$ | $0^{b}$ | $0^{b}$ |
| | 15 | 1,56x10$^{7a}$ | 5,02x10$^{6a}$ | $0^{b}$ | $0^{b}$ |
| | 18 | 1,14x10$^{7a}$ | 4,81x10$^{6a}$ | $0^{b}$ | $0^{b}$ |
| | 21 | 1,27x10$^{7a}$ | 1,82x10$^{6a}$ | $0^{b}$ | $0^{b}$ |

**Table S2**: Table evaluated fecal microbiome results and taxonomic groups with statistically significant differences separated by group p≤ 0.05.

| **Groups** | **Taxonomy** | **P value** | **Group with higher frequency** |
|---|---|---|---|
| **T21(Law_vs_Bra)** | **Family** | | |
| | Porphyromonadaceae | 0.012886830493507 | BRA |
| | Streptococcaceae | 0.012886830493507 | LAW |
| | **Genus** | | |
| | *Parabacteroides* | 0.012886830493507 | BRA |
| | *Streptococcus* | 0.012886830493507 | LAW |
| **T21(Co_vs_Bra)** | **Filo** | | |
| | Firmicutes | 0.012879064669346 | BRA |
| | Fusobacteria | 0.0355240368313795 | CO |
| | **Class** | | |
| | Clostridia | 0.0474483843880802 | BRA |
| | Fusobacteriia | 0.0355240368313795 | CO |
| | **Order** | | |

| | | | |
|---|---|---|---|
| | Clostridiales | 0.0409535667814036 | BRA |
| | Fusobacteriales | 0.0355240368313795 | CO |
| | Lactobacillales | 0.0351950848989481 | CO |
| | **Family** | | |
| | [Paraprevotellaceae] | 0.010705311873033 | CO |
| | Clostridiaceae | 0.00248543353268081 | BRA |
| | Fusobacteriaceae | 0.0355240368313795 | CO |
| | Lactobacillaceae | 0.0149386031285565 | CO |
| | **Genus** | | |
| | *[Prevotella]* | 0.012879064669346 | CO |
| | *Clostridium* | 0.010705311873033 | BRA |
| | *Fusobacterium* | 0.0355240368313795 | CO |
| | *Lactobacillus* | 0.0149386031285565 | CO |
| | *p-75-a5* | 0.0474483843880802 | CO |
| **T21(Co_vs_Law)** | **Filo** | | |
| | Firmicutes | 0.0256547699171202 | LAW |
| | Fusobacteria | 0.00631375918655783 | CO |
| | Proteobacteria | 0.0351950848989481 | CO |
| | **Class** | | |
| | Bacilli | 0.00153997525783769 | LAW |
| | Deltaproteobacteria | 0.012879064669346 | CO |
| | Epsilonproteobacteria | 0.00591269060812482 | CO |
| | Fusobacteriia | 0.00631375918655783 | CO |
| | **Order** | | |
| | Campylobacterales | 0.00591269060812482 | CO |
| | Desulfovibrionales | 0.010705311873033 | CO |
| | Enterobacteriales | 0.00830344690531291 | CO |
| | Fusobacteriales | 0.00631375918655783 | CO |
| | Lactobacillales | 0.000923824907537386 | LAW |
| | Pasteurellales | 0.0156848401759963 | CO |
| | **Family** | | |
| | [Odoribacteraceae] | 0.00143598783526406 | CO |
| | [Paraprevotellaceae] | 0.000923824907537386 | CO |
| | Bacteroidaceae | 0.00187902637312587 | CO |
| | Campylobacteraceae | 0.00342874651022508 | CO |
| | Desulfovibrionaceae | 0.010705311873033 | CO |
| | Enterobacteriaceae | 0.00830344690531291 | CO |
| | Fusobacteriaceae | 0.00631375918655783 | CO |
| | Lactobacillaceae | 0.0127092847550734 | LAW |
| | p-2534-18B5 | 0.0276173541564825 | LAW |
| | Pasteurellaceae | 0.0156848401759963 | CO |
| | Porphyromonadaceae | 0.00591269060812482 | CO |
| | Streptococcaceae | 0.0425377920562403 | LAW |

| | | | |
|---|---|---|---|
| | Veillonellaceae | 0.00594276612246729 | LAW |
| | **Genus** | | |
| | *[Eubacterium]* | 0.00279865510717577 | LAW |
| | *[Prevotella]* | 0.00196463024569095 | CO |
| | *Anaerovibrio* | 0.0256783417053815 | CO |
| | *Bacteroides* | 0.00187902637312587 | CO |
| | *Butyricimonas* | 0.00197139594296633 | CO |
| | *Campylobacter* | 0.00342874651022508 | CO |
| | *Catenibacterium* | 0.027559127653885 | CO |
| | *Desulfovibrio* | 0.0256547699171202 | CO |
| | *Dialister* | 0.010705311873033 | LAW |
| | *Fusobacterium* | 0.00631375918655783 | CO |
| | *Lactobacillus* | 0.00175250848921009 | LAW |
| | *Megasphaera* | 0.00248543353268081 | LAW |
| | *Odoribacter* | 0.00142985438326551 | LAW |
| | *Oscillospira* | 0.0351950848989481 | CO |
| | *p-75-a5* | 0.00153997525783769 | CO |
| | *Parabacteroides* | 0.00704657239034717 | CO |
| | *Shuttleworthia* | 0.0238202172795873 | LAW |
| | *Streptococcus* | 0.0149386031285565 | LAW |
| **T21(co_vs_neg21)** | **Filo** | | |
| | Fusobacteria | 0.00631375918655783 | CO |
| | **Class** | | |
| | Bacilli | 0.0301176534687101 | CO |
| | Deltaproteobacteria | 0.0474483843880802 | CO |
| | Fusobacteriia | 0.00631375918655783 | CO |
| | Opitutae | 0.0133160403375067 | NEG |
| | **Order** | | |
| | Desulfovibrionales | 0.00884804722804454 | CO |
| | Enterobacteriales | 0.00354574677030781 | CO |
| | Fusobacteriales | 0.00631375918655783 | CO |
| | Lactobacillales | 0.0256547699171202 | NEG |
| | Pasteurellales | 0.0156848401759963 | CO |
| | **Family** | | |
| | [Odoribacteraceae] | 0.00362001666753251 | CO |
| | [Paraprevotellaceae] | 0.0072736188296829 | CO |
| | Bacteroidaceae | 0.00860879727082779 | CO |
| | Christensenellaceae | 0.0072736188296829 | NEG |
| | Desulfovibrionaceae | 0.00884804722804454 | CO |
| | Enterobacteriaceae | 0.00354574677030781 | CO |
| | Fusobacteriaceae | 0.00631375918655783 | CO |
| | Lactobacillaceae | 0.0175054975400099 | NEG |
| | Pasteurellaceae | 0.0156848401759963 | CO |

| | | | |
|---|---|---|---|
| | **Genus** | | |
| | *[Eubacterium]* | 0.037417959614756 | NEG |
| | *[Prevotella]* | 7,77E-09 | CO |
| | *Anaerovibrio* | 0.0028415273840731 | CO |
| | *Bacteroides* | 0.00860879727082779 | CO |
| | *Butyricimonas* | 0.01045624760529 | CO |
| | *Clostridium* | 0.0474483843880802 | CO |
| | *Desulfovibrio* | 0.00594276612246729 | CO |
| | *Fusobacterium* | 0.00631375918655783 | CO |
| | *Lactobacillus* | 0.0127092847550734 | NEG |
| | *Megasphaera* | 0.0301176534687101 | NEG |
| | *Odoribacter* | 0.000422760407398187 | NEG |
| | *p-75-a5* | 0.00153997525783769 | CO |
| | *Shuttleworthia* | 0.0127092847550734 | NEG |
| **T21(law21_vs_neg21)** | **Class** | | |
| | [Lentisphaeria] | 0.0453003901882632 | NEG |
| | Bacilli | 0.037417959614756 | LAW |
| | **Order** | | |
| | Campylobacterales | 0.012837523673437 | NEG |
| | GMD14H09 | 0.00463155837210344 | LAW |
| | Lactobacillales | 0.0473071819821046 | NEG |
| | WCHB1-41 | 0.0298476426791938 | NEG |
| | **Family** | | |
| | Campylobacteraceae | 0.00523650755848898 | NEG |
| | p-2534-18B5 | 0.0359098003120898 | NEG |
| | Porphyromonadaceae | 0.0473071819821046 | NEG |
| | RFP12 | 0.0354526082163918 | NEG |
| | Streptococcaceae | 0.0213986841852967 | LAW |
| | Veillonellaceae | 0.0192307692307692 | LAW |
| | **Genus** | | |
| | *Campylobacter* | 0.0268002289808724 | LAW |
| | *Catenibacterium* | 0.037417959614756 | NEG |
| | *Dialister* | 0.00218478519730371 | LAW |
| | *Megasphaera* | 0.0156056085521693 | LAW |
| | *Streptococcus* | 0.012593249898966 | LAW |
| | *Unassigned* | 0.0225001812025638 | NEG |
| **T21(bra21_vs_neg21)** | **Genus** | | |
| | *Odoribacter* | 0.00962198825643798 | NEG |
